# Mini Review on Nanomaterial-Driven Substrate Optimization of Polyamide Thin-Film Nanocomposite Membranes for FO, RO and NF Applications

**DOI:** 10.3390/membranes16040131

**Published:** 2026-03-31

**Authors:** Shabin Mohammed, Ahmed Elmekawy, Ranwen Ou, Hanaa M. Hegab

**Affiliations:** 1 Faculty of Engineering, Higher Colleges of Technology, Abu Dhabi 25035, United Arab Emirates; aelmekawy@hct.ac.ae; 2 College of the Environment & Ecology, Xiamen University, Xiamen 361102, China; ouranwen@xmu.edu.cn

**Keywords:** TFC, nanomaterials, polyamide, FO, RO, NF, membrane

## Abstract

The rising demand for clean water has reinforced the importance of thin-film composite TFC polyamide membranes in desalination and wastewater treatment. While improvements often target the selective layer, these can sometimes reduce stability or selectivity. An alternative approach is to tailor the porous support, particularly through the incorporation of nanomaterials such as metal oxides, carbon-based nanomaterials, metal–organic frameworks (MOFs), zeolites, and cellulose-based materials, to improve overall membrane performance. The modification of membrane substrates through the incorporation of nanofillers has demonstrated notable advantages, including enhanced hydrophilicity, improved mechanical stability, and increased porosity. These improvements collectively contribute to higher permeability, reduced internal concentration polarization and enhanced separation performance in FO, NF, and RO applications. The review starts by clearly distinguishing substrate modification, in which nanomaterials are localized in the porous support, from interlayer modification, which involves constructing a distinct layer between the support and selective layer. This concise review highlights current developments in the nanomaterial-based support modification of polyamide TFC membranes; it summarizes nanomaterials selections, incorporation techniques, and resulting property changes. Current challenges and potential research opportunities are also discussed.

## 1. Introduction

Water scarcity has emerged as one of the most pressing environmental challenges of the 21st century, driven by the combined effects of urban expansion, industrial growth, and climate variability [1]. At the same time, governments around the world are tightening rules on water quality, which means that more reliable ways to treat it are urgently needed. Membrane purification processes have become central to this effort, offering compact, energy-efficient, and highly selective solutions for producing safe water [2]. Polymeric membranes hold a dominant position due to their moderately low production costs, structural versatility, and ease of large-scale manufacturing [3]. Their tunable chemical and physical properties facilitate the exact regulation of pore structure and surface features, allowing for the effective removal of pollutants in a wide range of applications. Furthermore, the dense molecular architecture of certain polymeric membranes provides exceptional selectivity at the nanoscale, making them indispensable in modern water and wastewater treatment systems.

**Figure 1:**
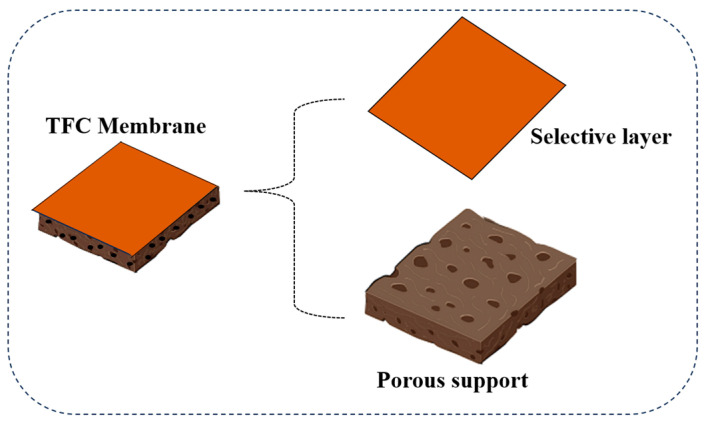
The structure of thin-film composite membranes: a thin selective layer is supported on a porous membrane support.

Thin-film composite (TFC) membranes represent one of the most widely adopted and well-established polymeric membrane configurations for high-performance water purification (Figure 1) [4]. They have a structure comprising a dense yet very thin selective layer that was created using interfacial polymerization on a porous support substrate. Most of the time, the selective layer is made of polyamide, which controls how effectively the membrane removes contaminants and the substrate provides the membrane with the mechanical stability it needs.

Many strategies have been developed over the years to improve the performance of TFC membranes. These include modifying the conditions of the interfacial polymerization process and changing the chemical structure of the selective layer [5]. The TFC design has a unique advantage since it is modular. This design allows each layer (substrate, interlayer, and selective layer) to be independently optimized for selectivity, permeability, and durability.

Given that the selective layer primarily governs separation efficiency, many studies have focused on modifying this component. However, such modifications can sometimes lead to adverse effects, including reduced cross-linking efficiency or the formation of interfacial voids, which diminish selectivity [6]. Similarly, while interlayer modifications have shown promise, concerns remain regarding their long-term stability [7]. As an alternative, researchers have increasingly explored the modification of the support layer prior to interfacial polymerization, often by incorporating functional nanomaterials [8,9,10]. The presence of nanofillers with diverse chemical and physical properties can significantly influence the surface and bulk characteristics of the substrate, which in turn affects the formation of the selective layer and the overall membrane performance.

This mini review focuses on recent progress in the modification of TFC membrane support layers using nanomaterials, aiming to enhance key performance parameters such as permeability, selectivity, fouling resistance, and mechanical robustness. In recent years, several reviews have addressed substrate modifications using a wide range of approaches, often grouping nanomaterial incorporation together with chemical treatments, grafting, or other modification strategies. This review places particular emphasis on nanomaterial- or filler-modified support layers to provide a more precise understanding of developments in this specific area. In addition to performance trends, the review discusses mechanistic insights into how substrate properties such as hydrophilicity and pore structure influence polyamide layer formation during interfacial polymerization. To ensure conceptual clarity, a distinction between interlayer modification and substrate modification is first provided. Subsequent sections discuss the effects of nanomaterial-based substrate modification on membrane properties and performance, before concluding with the challenges that remain and future research directions in this area.

## 2. Substrate vs. Interlayer Modification: Clarifying the Overlap

The distinction between substrate modification and interlayer engineering is often blurred, primarily due to the location and distribution of incorporated nanomaterials. Both methods attempt to improve membrane performance by enhancing interfacial properties, but they are different in how they are defined and used. Substrate modification typically involves altering the surface or bulk properties of the porous support prior to interfacial polymerization [10]. This can be achieved by blending nanomaterials into the casting solution or chemical grafting. These modifications enhance characteristics like hydrophilicity, roughness, and pore structure, thereby influencing the formation and quality of the polyamide (PA) selective layer. In contrast, interlayer engineering refers to the deliberate introduction of a distinct nanostructured layer between the substrate and the PA layer, usually after the support is fabricated [11]. This interlayer, which is applied using methods like vacuum filtering, spin coating, or layer-by-layer assembly, controls the diffusion of monomers and the shape of the PA layer, and adds new functions like antifouling or better mechanical stability.

The overlap between the two techniques generally comes from situations when nanomaterials are only localized on or near the surface of the substrate, creating a thin zone that acts like an interlayer. For example, nanomaterials introduced during substrate casting may move toward the surface, affecting interfacial polymerization in a way that is similar to an interlayer that was applied separately fabrication [12]. These hybrid structures, while not fitting cleanly into either category, exhibit features of both approaches. To address this ambiguity, our review clearly defines the scope based on the functional location and integration mode of the nanomaterials. We only include studies where nanomaterials are embedded within the substrate or localized at its surface without forming a separate, continuous interlayer. Studies that deliberately construct a distinct interlayer, regardless of nanomaterial use, are excluded. This focused criterion enables more precise analysis of substrate modification strategies while recognizing that these approaches may exist along a continuum in membrane interface design.

## 3. Nanomaterials for Substrate Modification

In the support layer modification of TFC membranes, a variety of inorganic nanomaterials have been incorporated to alter the structural and surface characteristics of the substrate. These fillers include nanoparticles such as metal oxides, zeolites, MOFs, cellulose-based nanomaterials, carbon nanotubes, and 2D materials, such as graphene derivatives and MoS_2_ nanosheets. The most widely adopted approach for incorporating nanofillers into membrane supports involves dispersing the selected nanomaterials into the polymer dope solution before the casting and phase inversion processes [13]. This approach helps spread the nanofillers evenly throughout the polymer matrix, making sure they are incorporated in both the surface and the bulk of the support structure. This kind of integration lets the fillers change both the surface and interior properties of the substrate. Instead of spreading nanomaterials throughout the bulk, another technique concentrates on putting them directly onto the surface of the support [14]. This method is based on the idea that the interfacial polymerization reaction, which makes the selective layer, happens mostly on the surface of the substrate. Because of this, the bulk properties of the support do not usually have much of an effect on the final separation performance. Surface-only modification also has the benefit of needing fewer nanomaterials to make the membrane, which makes it a more material-efficient choice than bulk blending in the polymer dope solution. Adding nanomaterials to membranes modifies their properties in several ways, all of which work together to make them better. The next several sections will summarize how these things affect the membranes’ physicochemical properties and overall performance.

### 3.1. Improved Hydrophilicity

**Figure 2:**
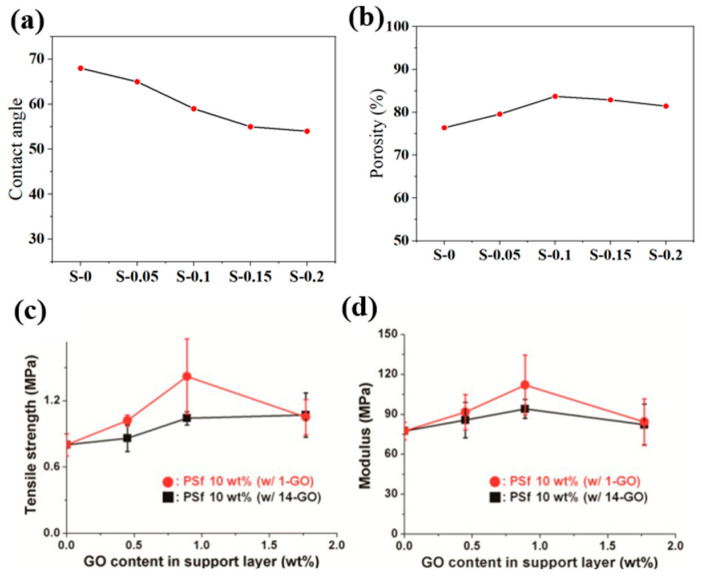
(**a**) Enhancement of hydrophilicity and (**b**) variations in porosity with the addition of nanozeolite in the membrane support, as reported in [12]. (**c**,**d**) Enhancement of mechanical strength with the addition of GO in membrane support, as observed in [23]. All figures have been reproduced with permission.

Adding nanofillers to the membrane support impacts how hydrophilic it is, especially when hydrophilic nanofillers are added (Figure 2a). For example, naturally hydrophilic nanozeolites significantly lower the water contact angle of the polysulfone (PSf) support (from about 68° to about 54° as the nanozeolite concentration rises), which makes the support more wettable [12]. Similarly, the addition of silica nanoparticles was also reported to reduce the contact angle considerably from ~74° to ~68° [15]. However, in some cases, this increment in hydrophilicity is not only limited within the substrate layer but also in terms of the overall wettability of the TFC membrane surface. For example, the presence of MXene in the support layer was reported to improve the overall hydrophilicity of the composite membranes [16]. While the improvement in overall membrane hydrophilicity is evident, it remains unclear whether this enhancement arises directly from the nanomaterial itself or from its influence on the interfacial polymerization process. Further investigation is required to clarify this mechanism.

Hydrophilic support permits piperazine (PIP) molecules to propagate evenly over the surface. This has an enormous impact on interfacial polymerization and, in final stages, controls the effectiveness of the membrane’s removal capacity. The hydrophilic nature of the membrane support promotes the formation of a thinner selective layer due to more controlled interfacial polymerization. In particular, a hydrophilic support can absorb and retain a greater amount of aqueous-phase amine monomers within its pores and on its surface, ensuring that the monomers are evenly distributed across the reaction interface [17]. This minimizes the occurrence of “dry spots” devoid of amine monomers, which could otherwise lead to defects or uneven polyamide layer formation. Furthermore, hydrophilic fillers attract and hold water molecules at the interface, which in turn slows the diffusion of amine monomers into the organic phase [14]. This moderated diffusion rate facilitates more uniform monomer transport, prevents excessively rapid polymer growth, and ultimately results in the formation of a thinner, smoother, and more defect-free selective layer. Lastly, hydrophilic surface forms a hydration layer, which acts as a physical and energetic barrier against foulants, thereby imparting fouling resistance [9].

### 3.2. Altered Porosity and Pore Size

The presence of nanoparticles in the polymer dope solution can also influence viscosity, hydrophilicity, and consequently the phase inversion process, leading to variations in pore characteristics (Figure 2b). For example, the nanoparticles’ increased hydrophilicity speeds up phase inversion and makes it happen right away, which makes the support more porous [18]. On the other hand, a higher viscosity in the solution may cause delayed de-mixing, which leads to substrates with less porosity. Consequently, the presence of nanofillers increases or decreases porosity based on the concentration at which they are used. Nanofillers such as GO, silica, MOFs and zeolites were demonstrated to alter the porosity based on their concentration [14,15,19,20]. For example, the addition of silica at optimum conditions rendered an increased porosity of ~67%, which was originally ~57% [15].

Changes in the surface and bulk pore structures of the support tend to be very important for the interfacial polymerization reaction because they transform the way the membrane is made as well as how it works [21]. For instance, the surface pore characteristics, in particular, play a critical role in the interfacial polymerization (IP) process, which forms the selective layer. The size and distribution of surface pores regulate the migration of amine monomers during interfacial polymerization reaction. Smaller pores limit the volume of aqueous phase retained at the surface, making the transport of monomers, such as PIP, predominantly diffusion driven [22]. Conversely, larger pores can retain more aqueous phase, facilitating the stronger convective transport of amine molecules to the organic phase. These differences in monomer transport dynamics ultimately affect the thickness, morphology, and cross-linking density of the polyamide selective layer, thereby influencing the overall performance of the membrane.

### 3.3. Enhanced Mechanical Strength

When it comes to membranes, their mechanical properties are quite important, especially in nanofiltration and reverse osmosis, where they must function under high pressures. Adding nanofillers could have a significant impact on the mechanical stability of the membrane support. The extent of their assistance depends on the intrinsic characteristics of the fillers. Nanofillers with huge surface areas and/or large aspect ratios can be particularly effective under optimized conditions for strengthening the support [4]. Their high aspect ratio and stiffness allow them to bear mechanical loads and transfer stress effectively when well dispersed within the polymer matrix, reducing the risk of structural collapse in the highly porous support (Figure 2c,d) [23]. A larger interfacial contact area between the nanofiller and polymer matrix facilitates improved load transfer over a wider region, even at relatively low filler concentrations [4]. Consequently, improved load transfer facilitates resistance to deformation and failure. However, to achieve such advantages, the nanofillers and the polymer matrix of the support layer must be compatible. Excellent adhesion between the polymer and the fillers helps stress to move between them more easily, which improves the structure’s strength and long-term mechanical stability.

It is also worth noting that, compared to the incorporation of nanomaterials within the polymer matrix, merely modifying the surface of a membrane or support with nanomaterials has a minimal impact on mechanical stability. The load transfer to the incorporated nanomaterial is more effective when the nanomaterial is dispersed uniformly within the matrix [24,25]. Therefore, surface modification has limited impact here, as nanomaterials are limited to a thin outer layer rather than the load-bearing bulk.

## 4. Recent Studies on the Modification Support Layers Using Nanomaterials

Nanomaterials have traditionally been incorporated into the selective layer of TFC membranes, the properties of which directly dictate separation performance. However, increasing attention has been paid to modifying the support layer, given its influence on interfacial polymerization and, consequently, on the morphology and properties of the formed selective layer. The following sections and Table 1, Table 2 and Table 3 summarize recent studies on support modification strategies specifically for nanofiltration, reverse osmosis, and forward osmosis membranes.

### 4.1. Forward Osmosis Membranes

**Figure 3:**
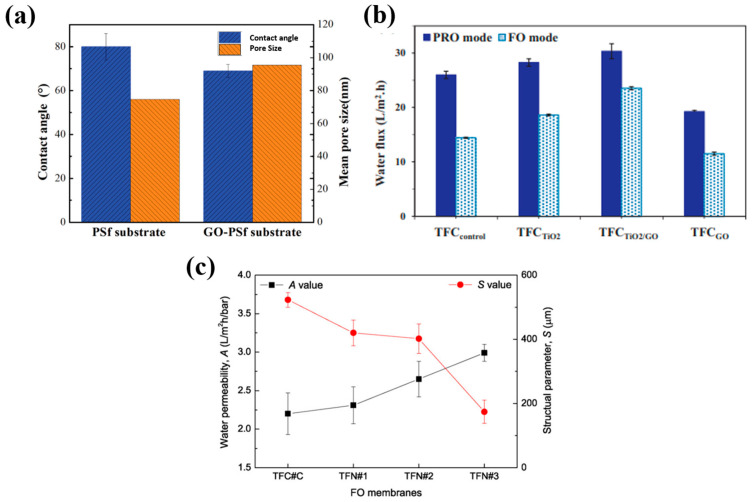
(**a**) Comparison of hydrophilicity and mean pore size of PSf supports from Yuan Li’s study [20]. The incorporation of GO markedly increased the substrate’s hydrophilicity and enlarged its mean pore size. (**b**) Water flux of TFC membranes with supports modified by TiO_2_ and GO. Results showed that the combined effect of GO and TiO_2_ gave the best performance [28]. (**c**) The effect of silica nanoparticles on the nanofibrous membrane support [30]. Higher silica nanoparticles decreased concentration polarization, leading to improved flux. All figures have been reproduced with permission.

Graphene oxide (GO), by itself or in combination with other nanomaterials, has been studied as an additive in forward osmosis membranes due to its hydrophilic nature and tunable surface chemistry [20,26,27]. GO was incorporated into the support layer by blending it with a polysulfone casting solution and forming the substrate via phase inversion [27]. This modification lowered the contact angle from 78.6° to 70.9°, increased porosity from 72.2% to 78.9%, and enlarged the mean pore size from 18.5 nm to 21.1 nm. These changes reduced the structural parameter and improved water flux in FO mode from 21.5 to 27.8 LMH, with a slight drop in reverse salt flux (5.1 to 4.7 gMH) and minor gains in salt rejection. Later, Yuan Li et al. compared the influence of GO in support and the influence of the selective layer of TFC membranes in forward osmosis, where GO was incorporated into the support layer in a similar fashion [20]. The GO–polysulfone substrate demonstrated improved hydrophilicity (contact angle reduced from 80° to 69°), larger average pore size (95.5 nm vs. 74.6 nm), and higher porosity (~55% vs. ~36%) than the pristine substrate, owing to GO’s hydrophilic functional groups and its influence on the solvent–nonsolvent exchange rate, as presented in Figure 3a. These structural changes enhanced water permeability, increasing water flux from 15.6 to 28.5 LMH and slightly lowering reverse salt flux, with a modest rise in salt rejection. The main reason for this improvement was that the more porous hydrophilic support structure made it easier for water to pass through and diminished internal concentration polarization. However, the biofouling resistance remained largely unchanged from the unmodified TFC membrane and less significant than active-layer modification. Based on the results obtained, authors concluded that GO incorporation into the selective-layer minimized cell adhesion and subsequent biofilm growth due to its significantly higher hydrophilicity relative to the support layer modification.

Researchers investigated the use of dual-nanomaterial systems with GO to make membrane support much stronger [28,29]. For example, Sirinupong et al. reported that incorporating both TiO_2_ and GO into a PSf support significantly improved FO performance [28]. With 0.5 M NaCl as the draw solution, water flux increased from 5.9 LMH for the control to 9.8 LMH with TiO_2_ and 12.3 LMH with TiO_2_/GO (Figure 3b). TiO_2_ improved hydrophilicity and created long finger-like pores, while GO contributed additional functional groups that enhanced wettability and water transport pathways. The combined modification outperformed either additive alone, whereas GO-only supports exhibited irregular pores that hindered flux. A similar synergistic effect was observed for the GO–layered double hydroxide (LDH) combination, where GO enhanced salt rejection and promoted the uniform dispersion of LDH nanosheets in the PSf matrix, mitigating the aggregation typically seen with LDH alone, while LDH improved hydrophilicity and pore development, thereby increasing permeability [29].

Metal oxides like silica and alumina nanoparticles are good additions for FO membrane supports because they naturally attract water due to their hydrophilic properties [15,30,31]. Incorporating silica nanoparticles into nanofibrous supports rendered a huge surface more wettable with higher porosity, and reduced the internal concentration polarization, which led to a higher water flux (Figure 3c) [30]. Comparable improvements have been reported with zeolites [32], and, more recently, ZIF-8 [33]. ZIF-8 could alter both the polyamide active layer and the PSf support for purifying aquaculture effluent. The separation of pharmaceutical contaminants showed that they performed better in tandem. In the PA layer, ZIF-8 forms hydrogen bonds with amide groups to make the surface more hydrophilic and improve its morphology. In the PSf layer, it coordinates with sulfone groups to improve pore structure and lower internal concentration polarization [33]. The dual-layer incorporation of ZIF-8 maximized these effects, delivering higher water flux, reduced reverse salt flux, and superior contaminant rejection compared to single-layer modifications.

**Table 1:** Comparison of TFC-FO membranes incorporating nanomaterials.

Nanomaterial	Method of Addition	Performance (Optimal)	Impact on Substrate & Performance	Ref.
Al_2_O_3_	Blended in PSf substrate (0.5%) and in PA layer (0.05%)	Water flux ≈ 27.6 LMH (DI/1 M NaCl), reverse salt flux ≈ 7.1 g/m^2^ h	Enhanced flux more than single-layer addition; substrate and PA more hydrophilic/porous, lowering ICP. Membrane remained stable long-term.	[31]
GO	GO blended into PSf substrate (0.15%) and added to PA layer (100 ppm)	Flux up to ≈31.8 LMH (dual modification) vs. ≈22.4 LMH (PA only) and 28.5 (substrate only); salt rejection ~94%	GO in substrate increases porosity (≈55% vs. 36% pristine) and decreases contact angle, boosting flux.	[20]
GO	Blended in PSf substrate via phase inversion	Flux ≈ 19.8 LMH (AL-FS) vs. 6.08 LMH (blank); reverse-flux selectivity 5.75 L/g vs. 3.36 (blank)	Added GO makes PSf support thinner, more porous and hydrophilic. This lowers structural parameters and allows better PA formation, yielding ≈3× higher flux.	[27]
Zeolite (clinoptilolite)	Blended in PES substrate via phase inversion	FO/PRO flux ~2.74 LMH (optimal) vs. 1.93 LMH (control), a 43% increase; NaCl rejection ≈ 94.7%	Zeolite raises substrate surface porosity and reduces S (0.78 → 0.48 mm), greatly reducing ICP. Resulting TFN membrane shows much higher water flux without sacrificing rejection.	[32]
ZIF-8 MOF	Added to PA layer (0.025%, in TMC solution) and in situ in PSf substrate (up to ~2%)	PA-only-modified: flux ≈ 11.3 LMH (2.3× the pristine 4.9 LMH); high PPCP rejection (>90%)	ZIF-8 makes PA surface more hydrophilic, increasing permeability. Dual-layer-modified membrane achieved high water recovery (≈89% in 3.5 days).	[33]
LDH/GO hybrid nanosheets	Blended (2 wt%) into PSf substrate	FO flux ≈ 13.4 LMH (DI/1 M NaCl); PRO flux ≈ 23.6 LMH; very low reverse flux (~6.2 g/m^2^ h)	LDH/GO increases support porosity and hydrophilicity. The TFN-2 membrane had a low S (~0.138 mm), so significantly higher permeability and reverse-selectivity than unmodified.	[29]
SiO_2_	Blended into PSf substrate	FO flux increased from 9.1 to 22.3 LMH (AL-FS) and 18.2 to 41.9 LMH (AL-DS) at 2 M NaCl;	SiO_2_ improves substrate wettability and porosity, lowering ICP. Very high loadings (5%) reduce salt rejection (NaCl rejection drops from ~87% to 78%).	[15]
TiO_2_ + GO	Blended into PSf substrate (equal 0.5% each)	FO flux ~12.3 LMH (0.5 M NaCl, AL-FS) vs. 5.9 LMH (control), slight reduction in rejection (~90% vs. 96%)	TiO_2_/GO combination yields the lowest S (≈0.20 vs. 0.31 control). Substrate contact angle was lowest, improving water uptake; PA layer became thinner/rougher. Overall permeability doubled with small sacrifice in rejection.	[15]
SiO_2_	Embedded in electrospun PEI nanofiber substrate	FO flux ≈ 42 LMH (AL-FS) and 72 LMH (AL-DS) with 1.0 M NaCl	Silica increased substrate porosity (to ~83%) and pore size; structural parameter S dropped to ~0.174 mm, achieving highest FO flux reported.	[30]
MXene	Blended in the support layer	FO flux ~13.63 LMH	Enhanced hydrophilicity reduced internal concentration polarization.	[16]

Recently, there has been growing interest in utilizing novel 2D materials, particularly MXene, for membrane fabrication and modification [34]. While most of this modification of TFC has been done on the selective or intermediate layer, Y. Wang et al. investigated the influence of TFC structure and performance due to the incorporation of MXene into the support layer in FO membranes [16]. It was reported that modification resulted in improved water flux, stemming from the reduced internal concentration polarization and enhanced structural morphology. Although MXene-based membranes have demonstrated promising performance in recent years, their long-term structural stability remains a concern. MXenes are prone to oxidation in the presence of dissolved oxygen, which can lead to the gradual degradation and deterioration of membrane performance [35]. Therefore, further studies are needed to enhance and verify the stability of MXene-based membranes under practical operating conditions.

### 4.2. Reverse Osmosis Membranes

Materials investigated for RO membrane modification include graphene oxide (GO), metal–organic frameworks (MOFs), and cellulose nanocrystals (CNCs). These nanomaterials exert diverse influences on membrane formation and performance, which are discussed in the subsequent sections. Overall, these studies indicate that different nanomaterials contribute in distinct ways, with GO primarily improving mechanical stability and hydrophilicity, MOFs influencing the pore structure and selective-layer morphology, and cellulose-based fillers offering better compatibility.

**Figure 4:**
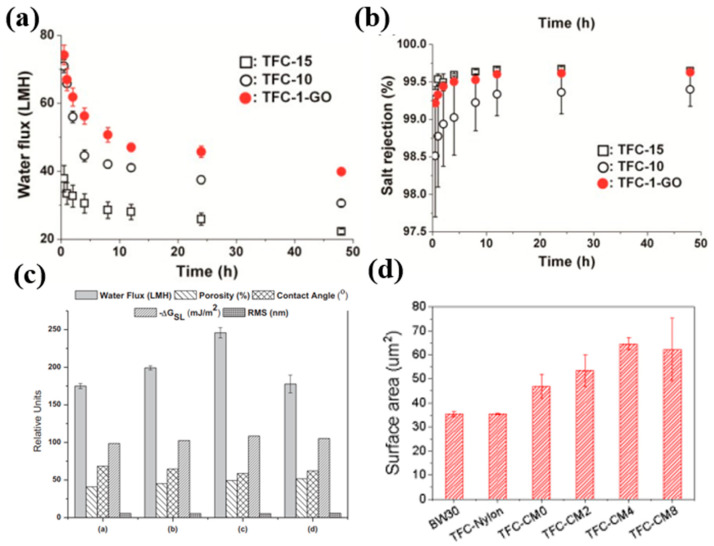
(**a**) Water flux and (**b**) salt rejection exhibited by membranes fabricated on GO-modified substrates. Water flux was enhanced significantly without affecting the membrane selectivity [23]. (**c**) Enhancements in membrane properties by incorporating MOFs into the substrate layer [19]. (**d**) Increase in effective filtration area with the addition of MOFs into the fibrous support layer due to hilly morphology [37]. All figures have been reproduced with permission.

Exfoliated graphene oxide nanosheets were initially utilized to augment the mechanical stability of the membrane support [23]. Lowering the polymer concentration during phase inversion rendered the PSf support more porous, which facilitated water flow, but it also made the structure less robust. The incorporation of 0.9 wt% single-layer GO into a 10 wt% PSf support layer successfully offset this limitation, enabling high porosity without compromising mechanical robustness. The GO concentration was insufficient to substantially affect interfacial polymerization, as seen by minimal alterations in active-layer characteristics: surface roughness, cross-linking degree indicated by the N/O ratio, and active-layer thickness. As a result, membrane selectivity remained unchanged, while water flux increased significantly (Figure 4a,b). This suggests that GO-based substrate modification is particularly effective for enhancing permeability while preserving RO selectivity.

Later, the same group investigated the synergistic effects of GO in both the support and selective layer [36]. Adding GO to the support layer altered its surface and structure significantly, even if selective-layer modification was the primary approach used to improve chlorine and fouling resistance. The contact angle dropped from 59° to 42°, which meant the surface was more hydrophilic, and the surface charge (measure by zeta potential) altered from relatively positive to considerably negative. The hydrophilic support additionally assisted in the GO nanosheets dispersing evenly across the polyamide layer, which prevented them from aggregating together and creating smooth, low-roughness areas. These improvements indirectly increased water flow and maintained the surface roughness at an ideal level, thereby improving the overall performance of the membrane. Compared with support only modification, this dual-layer approach offers additional functional benefits but comes with increased fabrication complexity.

In 2017, Park et al. reported that incorporating acid-treated HKUST-1 MOFs into the polysulfone support enhanced the membrane’s hydrophilicity and porosity, leading to macrovoid formation and improved surface properties [19]. These adjustments made it feasible for a thinner and smoother polyamide layer to form during interfacial polymerization, boosting water flux and fouling resistance without sacrificing salt rejection, as seen in Figure 4c. In contrast to GO, MOF-based support mainly affects RO performance by tailoring substrate porosity and polyamide layer formations.

Liu et al. revealed a novel way to add MOFs to the membrane support. In their study, the support was formed entirely of cellulose nanofibers (CNFs) [37]. They created a homogeneous hybrid support by mixing MOF particles directly into the CNF dispersion before vacuum filtration. This approach created a mechanically strong and very porous substrate that stuck well to the polyamide (PA) layer. The CNF–MOF support facilitated the growth of a “hilly” PA surface morphology, providing an enhanced filtration area (Figure 4d). This morphology, combined with the hydrophilicity imparted by CNFs and MOFs, resulted in high water permeability and excellent salt rejection. This hybrid strategy combines the structural advantages of MOFs with the compatibility and mechanical stability of cellulose-based supports.

Cellulose-based materials have also been incorporated into PSf supports by dispersing them directly into the casting solution. A clear comparison between microcrystals and nanocrystals emphasized the advantages of the nanoscale dimension [38]. While cellulose microcrystals mainly improved the bulk strength of the support, their lower surface area limited their ability to influence pore structure and surface chemistry. In contrast, cellulose nanocrystals, with their higher aspect ratio and greater surface area, markedly enhanced hydrophilicity, pore morphology, and surface properties, making them more effective modifiers than microcrystals. From a practical standpoint, cellulose-based fillers offer advantages in cost, compatibility, and scalability, even though their performance gains are often more moderate than those of inorganic nanomaterials.

**Table 2:** Comparison of TFC-RO membranes incorporating nanomaterials.

Nanomaterial	Method of Addition	Performance (Optimal)	Impact on Substrate & Performance	Ref.
Carboxylated Cellulose nanocrystals (CNC)	Added to PA active layer vs. in support (two configurations)	PA layer: higher flux; Support layer: higher salt rejection (≈96% → 98%+)	CNC in the PA layer enhanced hydrophilicity and flux, while CNC in the support layer produced a denser PA layer with higher salt rejection and strength.	[39]
HKUST-1 (Cu-BTC) MOF	Blended into PSf support; interfacial polymerization of MPD/TMC on top.	Water flux ~245.8 LMH (vs. 175 LMH for bare PSf); NaCl rejection ≈98%; improved fouling resistance.	HKUST-1 enhanced support porosity and hydrophilicity, leading to a thinner PA layer and higher flux, while maintaining ~98% salt rejection and improved fouling resistance.	[19]
Cellulose nanofibers (CNF) + NH_2_-MIL-53(Al) MOF	Incorporated in the support through vacuum filtration	Water permeance 5.55 LMH/bar; NaCl rejection ~94.8%. Very high pressure (~22 bar) operation.	The CNF/MOF interlayer induced a crumpled, high-surface-area PA morphology, markedly enhancing water flux without compromising salt rejection, while maintaining mechanical stability under high pressure.	[37]
Cellulose nanocrystals (CNC) and microcrystals (CMC)	CNC blended into PSf support (phase inversion);	Support-layer CNC: ≈+40% water flux; NaCl rejection ≈ 95% (vs. ~95% baseline).	CNC in the support significantly increased hydrophilicity and porosity, enhancing flux with minimal effect on rejection.	[38]
GO	Dual-layer embedding: GO dispersed in PSf support casting solution and in selective layer	Water permeability +19% (vs. GO only in PA); Salt rejection ~99% (highest of all test membranes). Anti-biofouling +77%.	GO in both layers enhanced hydrophilicity and surface charge, increased flux with ~99% NaCl rejection, reduced fouling, and improved chlorine/biofouling resistance without flux loss.	[36]

### 4.3. Nanofiltration Membranes

A variety of nanomaterials have been employed to modify the substrates of thin-film composite nanofiltration membranes, including silica (SiO_2_) and titania (TiO_2_) nanoparticles [40], functionalized graphene oxide [41,42], carboxylated cellulose nanocrystals [39], magnesium hydroxide (Mg(OH)_2_) nanosheets [43], nanozeolites [13], and molybdenum disulfide (MoS_2_) nanosheets [44]. Among these, the mere incorporation of nanoparticles into the substrate layer provided similar enhancements in membrane properties and overall performance. For instance, the incorporation of metal oxides (SiO_2_ and TiO_2_), nanozeolites, molybdenum disulfide (MoS_2_) nanosheets, amine-functionalized graphene oxide, and Mg(OH)_2_ nanosheets into the membrane substrate enhanced hydrophilicity, increased negative surface charge, and reduced surface roughness, collectively contributing to improved interfacial polymerization, higher permeability, and superior antifouling performance.

**Figure 5:**
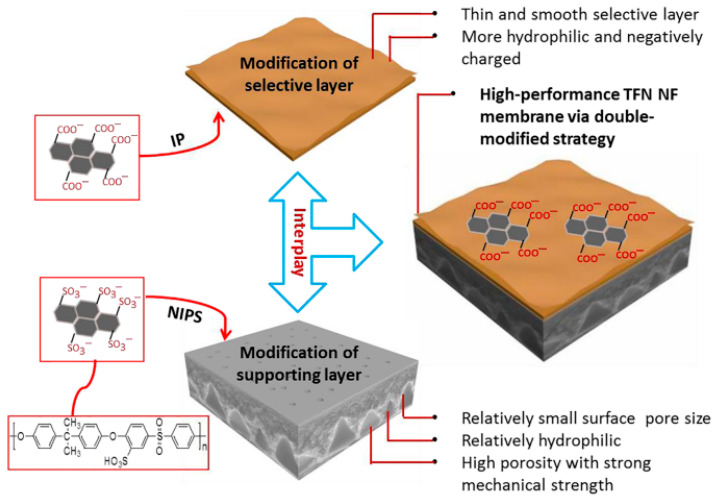
A schematic illustration of the dual modification strategy presented. MAH-GO in the selective layer and SG in the substrate synergistically enhanced the overall membrane performance. Reproduced with permission from [41].

A dual modification strategy was employed using functionalized graphene oxide derivatives, where maleic anhydride-modified GO (MAH-GO) was incorporated into the support layer and sulfonated graphene (SG) was dispersed into the aqueous phase for selective-layer formation [41]. The maleic anhydride groups in the substrate introduced additional reactive sites that bonded with amine monomers during interfacial polymerization, thereby enhancing the hydrophilicity, adhesion, and crosslinking density of the polyamide layer. In parallel, the sulfonyl groups of sulfonated graphene imparted strong hydrophilicity and a higher negative surface charge to the selective layer, improving ion rejection, antifouling resistance, and the smoothness of the film. Together, this dual modification produced a synergistic effect, significantly enhancing permeability while maintaining excellent salt rejection and fouling resistance, as illustrated in Figure 5. For instance, water flux increased from 32.4 LMH for pristine PPA-TFC to 64.3 LMH for SG-modified TFC, and further to 85.8 LMH for the dual-modified (SG + MAH-GO) membranes, all while maintaining high salt rejection at a 6-bar operating condition.

Yatao Liu et al. conducted a systematic investigation into the individual effects of substrate and selective-layer alteration to elucidate the role of nanofiller position [34,39]. Incorporating carboxylated cellulose nanocrystals into the substrate facilitated greater water permeability and selective-layer modification, while improving both permeability and selectivity at the same time. This could be attributed to the carboxyl groups facilitating tighter crosslinking and generating high electrostatic repulsion. These results indicate that the integration of nanoparticles into the support layer has a slight influence as it is less susceptible to the interfacial polymerization reaction that controls selective-layer formulation. The selective layer essentially determines the RO and NF membranes’ separation capacities. The substrate only influences the polymerization process. As a result, the standard method of incorporating nanomaterials into the polymer dope solution followed by inverting the phase usually only leads to slight enhancements. Recent research on nanozeolites showed this constraint even more clearly. It showed that modifying the surface of the substrate with dopamine contributed to far larger improvements compared with the earlier method of integrating it with bulk substrate [6,13]. Surface modification of the TFC support with nanozeolites not only improved hydrophilicity and surface charge (measure by zeta potential) but also induced a wrinkled morphology in the selective layer owing to the rugged reaction interface and controlled amine diffusion [14]. Consequently, the altered membrane demonstrated approximately 20% enhancement in effective filtration performance relative to the unaltered one, a characteristic not previously reported in NF membranes. This structural advantage translated into remarkable water permeance at 22.5 ± 2.2 LMH/bar, while maintaining high separation performance. Recently, hierarchical flower-like molybdenum disulfide (HF–MoS_2_) was utilized to modify the substrate layer for solvent-resistant nanofiltration membranes [44]. Enhancement was confined to the permeability largely along with slightly improved antifouling properties, similar to trends seen in aqueous systems. However, authors could not demonstrate any improvement in the solvent resistance of the membrane.

**Table 3:** Comparison of TFC-NF membranes incorporating nanomaterials.

Nanomaterial	Method of Addition	Performance (Optimal)	Impact on Substrate & Performance	Ref.
Magnetite-decorated sulfonated graphene oxide (MGO/SGO)	Blended into polysulfone (PSf) substrate (phase inversion); loose PA layer formed via electrospray IP.	Water flux ~44.4 LMH/bar; NaCl rejection ~96.1%; Congo red rejection 99.0%. High dye/salt selectivity (CR rejection >98%)	Embedded MGO/SGO greatly increased substrate porosity and hydrophilicity (enhancing flux). Negatively charged GO and Fe_3_O_4_ provide adsorption sites, boosting dye rejection and antifouling (FRR ≈ 99%).	[45]
Sulfonated graphene (SG) + MAH-GO nanofillers	SG blended in PSU support; MAH-GO incorporated in PA layer	Flux 85.8 LMH (264% of control); Na_2_SO_4_ rejection maintained	SG in PSU support increased porosity and hydrophilicity (defect-free, high-flux support); MAH-GO in PA yielded a thinner, smoother active layer, boosting flux (to 85.8 LMH) and antifouling without sacrificing rejection.	[41]
PDA-coated nanozeolite	PDA-zeolite layer on PSf support (pre-IP interlayer)	Flux 22.5 LMH/bar; Na_2_SO_4_ rejection 99.5%	Zeolite interlayer induced highly wrinkled PA morphology (increase surface area) and increased support hydrophilicity; membrane permeance tripled vs. pristine (≈22.5 LMH/bar) while preserving very high salt rejection.	[14]
Mg(OH)_2_ nanosheets (2D layers)	Blended in PSf support (phase inversion)	Flux ↑ ~196%; Na_2_SO_4_ rejection 97.7%	Mg(OH)_2_ increased support hydrophilicity and created 2D nanochannels; the PA layer became lighter and rougher (more linear), resulting in ~3× flux increase (196%) with ~97.7% Na_2_SO_4_ rejection and excellent fouling resistance (92.8% BSA FRR).	[43]
Amine-functionalized graphene oxide (NGO)	Blended in PSf substrate (phase inversion)	Flux ↑ 26.9% (to 49.3 LMH); Na_2_SO_4_ ~94.5%	NGO increased substrate pore size and hydrophilicity; this produced a much thinner PA layer (amino groups reacted with TMC) and thus higher flux (~49.3 LMH, +27%) with negligible change in salt rejection (Na_2_SO_4_ ~94%).	[42]
SiO_2_/TiO_2_ nanoparticles	Blended in PAN/PAN–PVDF support (phase inversion)	Flux ↑ (TiO_2_ best); high divalent/monovalent selectivity (MgSO_4_/NaCl ~4.63)	Enhanced substrate hydrophilicity and roughness, yielding thinner PA and higher flux; TiO_2_-modified supports achieved the highest flux and MgSO_4_/NaCl selectivity (~4.63).	[40]
Hierarchical flower-like MoS_2_ (HF–MoS_2_)	Blended in PVDF support (phase inversion)	Flux ~21.5 LMH/bar; Na_2_SO_4_ 98.6%	The substrate more porous, hydrophilic, and rougher; the PA layer became thinner (~56 nm) and more crosslinked, resulting in high flux (21.5 LMH/bar) and salt rejection (~98.6%), with excellent long-term stability and antifouling.	[44]
Nanozeolite (ball-milled zeolite)	Blended in PSf support (phase inversion)	Flux 17.1 LMH/bar; Na_2_SO_4_ 95.1%	Nanozeolite increased support hydrophilicity and porosity; membrane flux was high (17.1 LMH/bar) with very high MgSO_4_/Na_2_SO_4_ rejection (~94–95%)	[12]

## 5. Conclusions: Challenges and Future

The structural and physicochemical characteristics of the support layer in TFC membranes play an important role in shaping the formation, morphology, and quality of the polyamide selective layer, and thus the overall separation performance. Incorporating nanofillers into the support has emerged as a promising strategy to tune membrane properties without directly disturbing the selective layer, unlike active-layer modifications. However, the impact of substrate modification remains somewhat limited, as the support influences performance only indirectly in the interfacial polymerization process that governs selective-layer development. In pressure-driven RO and NF membranes, where separation performance is primarily dictated by the polyamide layer, improvements from the substrate are largely restricted to changes in influences monomer diffusion and crosslinking during film formation. As a result, traditional approaches that rely on dispersing nanomaterials into the casting solution prior to phase inversion generally provide only marginal improvements.

By contrast, in FO membranes, the role of support becomes more pronounced, as internal concentration polarization exerts a critical influence on water flux, allowing substrate modification to deliver more substantial performance enhancements. This highlights the necessity of customizing modification strategies to both the specific type of nanomaterial and its dispersion, as well as the envisioned separation process. With regard to the large-scale adoption of these strategies, several challenges exist in membrane development. Among these, achieving scalability in fabrication methods that ensure uniform nanofiller distribution remains the most critical challenge for commercialization. When fabrication is scaled up, variations in nanomaterial properties and processing conditions often make it difficult to maintain consistent membrane performance. Moreover, the leaching tendency of nanofillers requires further investigation at both laboratory and industrial scales, as this aspect has rarely been addressed in the literature. In addition, the production cost of nanomaterials also warrants attention. Although several studies report relatively inexpensive bio-based nanomaterials, particularly cellulose-based fillers with good cost and availability, recently introduced MOFs, zeolites, and other novel 2D materials remain costly and are still at an early stage of large-scale implementation.

To move this field forward, future studies should place greater emphasis on practical solutions such as green and low-cost routes for nanomaterials synthesis, covalent anchoring to limit nanofiller leaching, and in situ growth of nanofillers within the support matrix in order to achieve better compatibility. From a manufacturing perspective, bulk blending during phase inversion remains the most straightforward and scalable modification route, despite the fact that it typically yields only modest performance enhancements. Collectively, these strategies could improve membrane durability and more reproducible performance during scale up. These advances are essential for translating laboratory-scale improvements in permeability, fouling resistance, and mechanical stability into commercially viable nanocomposite TFC membranes with engineered support layers.

## Data Availability

No new data were created or analyzed in this study.

## References

[1] He C., Liu Z., Wu J., Pan X., Fang Z., Li J., Bryan B.A. (2021). Future global urban water scarcity and potential solutions. Nat. Commun..

[2] Yusuf A., Sodiq A., Giwa A., Eke J., Pikuda O., De Luca G., Di Salvo J.L., Chakraborty S. (2020). A review of emerging trends in membrane science and technology for sustainable water treatment. J. Clean. Prod..

[3] Seah M.Q., Chua S.F., Ang W.L., Lau W.J., Mansourizadeh A., Thamaraiselvan C. (2024). Advancements in polymeric membranes for challenging water filtration environments: A comprehensive review. J. Environ. Chem. Eng..

[4] Mohammed S., Aburabie J., Hashaikeh R. (2024). A review on the potential of cellulose nanomaterials for the development of thin film composite polyamide membranes for water treatment. Chemosphere.

[5] Saleh E.A.M., Kumar A., Alghazali T., Ganesan S., Shankhyan A., Sharma G.C., Naidu K.S., Rahbari-Sisakht M. (2025). Recent advances in thin film composite (TFC) membranes development: Materials and modification methods. Environ. Sci. Water Res. Technol..

[6] Wu H., Zhang Q., Xu B., Liu X., Lin Y., Jiang N., Yao H., Tang Y., Wang L., Yu L. (2024). In-situ interfacial polymerization of polyamide TFN membranes by adding a diamino-silane coupling agent: Toward enhanced desalination performance. Desalination.

[7] Yang Z., Sun P., Li X., Gan B., Wang L., Song X., Park H., Tang C.Y. (2020). A critical review on thin-film nanocomposite membranes with interlayered structure: Mechanisms, recent developments, and environmental applications. Environ. Sci. Technol..

[8] Wang L., Kahrizi M., Lu P., Wei Y., Yang H., Yu Y., Wang L., Li Y., Zhao S. (2021). Enhancing water permeability and antifouling performance of thin–film composite membrane by tailoring the support layer. Desalination.

[9] Zhang Q., Zhou R., Peng X., Li N., Dai Z. (2023). Development of support layers and their impact on the performance of thin film composite membranes (TFC) for water treatment. Polymers.

[10] Akbar Heidari A., Mahdavi H. (2023). Recent advances in the support layer, interlayer and active layer of TFC and TFN organic solvent nanofiltration (OSN) membranes: A review. Chem. Rec..

[11] Liu M., Zhang L., Geng N. (2023). Effect of interlayer construction on TFC nanofiltration membrane performance: A review from materials perspective. Membranes.

[12] Mohammed S., Nassrullah H., Aburabie J., Hashaikeh R. (2022). Fabrication of Thin Film Composite Membranes on Nanozeolite Modified Support Layer for Tailored Nanofiltration Performance. Membranes.

[13] Bărdacă Urducea C., Nechifor A.C., Dimulescu I.A., Oprea O., Nechifor G., Totu E.E., Isildak I., Albu P.C., Bungău S.G. (2020). Control of nanostructured polysulfone membrane preparation by phase inversion method. Nanomaterials.

[14] Mohammed S., Aburabie J., Hashaikeh R. (2023). Facile morphological tuning of thin film composite membranes for enhanced desalination performance. npj Clean Water.

[15] Huang Y., Jin H., Yu P., Luo Y. (2016). Polyamide thin-film composite membrane based on nano-silica modified polysulfone microporous support layer for forward osmosis. Desalination Water Treat..

[16] Wang Y., Nie Y., Chen C., Zhao H., Zhao Y., Jia Y., Li J., Li Z. (2022). Preparation and characterization of a thin-film composite membrane modified by mXene nano-sheets. Membranes.

[17] Dai R., Li J., Wang Z. (2020). Constructing interlayer to tailor structure and performance of thin-film composite polyamide membranes: A review. Adv. Colloid Interface Sci..

[18] Abbas T.K., Rashid K.T., Alsalhy Q.F. (2022). NaY zeolite-polyethersulfone-modified membranes for the removal of cesium-137 from liquid radioactive waste. Chem. Eng. Res. Des..

[19] Park H.M., Jee K.Y., Lee Y.T. (2017). Preparation and characterization of a thin-film composite reverse osmosis membrane using a polysulfone membrane including metal-organic frameworks. J. Membr. Sci..

[20] Li Y., Yang Y., Li C., Hou L. (2019). Comparison of performance and biofouling resistance of thin-film composite forward osmosis membranes with substrate/active layer modified by graphene oxide. RSC Adv..

[21] Liang X., Wang P., Wang J., Zhang Y., Wu W., Liu J., Van der Bruggen B. (2019). Zwitterionic functionalized MoS2 nanosheets for a novel composite membrane with effective salt/dye separation performance. J. Membr. Sci..

[22] Liu F., Wang L., Li D., Liu Q., Deng B. (2019). A review: The effect of the microporous support during interfacial polymerization on the morphology and performances of a thin film composite membrane for liquid purification. RSC Adv..

[23] Lee J., Jang J.H., Chae H., Lee S.H., Lee C., Park P., Won Y., Kim I. (2015). A facile route to enhance the water flux of a thin-film composite reverse osmosis membrane: Incorporating thickness-controlled graphene oxide into a highly porous support layer. J. Mater. Chem. A.

[24] Musa A.A., Bello A., Adams S.M., Onwualu A.P., Anye V.C., Bello K.A., Obianyo I.I. (2025). Nano-enhanced polymer composite materials: A review of current advancements and challenges. Polymers.

[25] Fu S., Sun Z., Huang P., Li Y., Hu N. (2019). Some basic aspects of polymer nanocomposites: A critical review. Nano Mater. Sci..

[26] Mohammed S. (2022). Graphene oxide: A mini-review on the versatility and challenges as a membrane material for solvent-based separation. Chem. Eng. J. Adv..

[27] Park M.J., Phuntsho S., He T., Nisola G.M., Tijing L.D., Li X., Chen G., Chung W., Shon H.K. (2015). Graphene oxide incorporated polysulfone substrate for the fabrication of flat-sheet thin-film composite forward osmosis membranes. J. Membr. Sci..

[28] Sirinupong T., Youravong W., Tirawat D., Lau W.J., Lai G.S., Ismail A.F. (2017). Synthesis and characterization of thin film composite membranes made of PSF-TiO2/GO nanocomposite substrate for forward osmosis applications. Arab. J. Chem..

[29] Lu P., Liang S., Zhou T., Mei X., Zhang Y., Zhang C., Umar A., Wang Q. (2016). Layered double hydroxide/graphene oxide hybrid incorporated polysulfone substrate for thin-film nanocomposite forward osmosis membranes. RSC Adv..

[30] Tian M., Wang Y., Wang R., Fane A.G. (2016). Synthesis and characterization of thin film nanocomposite forward osmosis membranes supported by silica nanoparticle incorporated nanofibrous substrate. Desalination.

[31] Ding W., Li Y., Bao M., Zhang J., Zhang C., Lu J. (2017). Highly permeable and stable forward osmosis (FO) membrane based on the incorporation of Al_2_O_3_ nanoparticles into both substrate and polyamide active layer. RSC Adv..

[32] Salehi T.M., Peyravi M., Jahanshahi M., Lau W., Rad A.S. (2018). Impacts of zeolite nanoparticles on substrate properties of thin film nanocomposite membranes for engineered osmosis. J. Nanopart. Res..

[33] Lin Y., Zheng N., Chen Y., Chang C. (2024). Incorporation of zeolitic imidazolate framework-8 (ZIF-8) in the polyamide and polysulfone layers of forward osmosis membranes for enhancing aquaculture wastewater recovery and PPCPs removal. J. Water Process. Eng..

[34] Karahan H.E., Goh K., Zhang C., Yang E., Yıldırım C., Chuah C.Y., Ahunbay M.G., Lee J., Tantekin-Ersolmaz Ş.B., Chen Y. (2020). MXene materials for designing advanced separation membranes. Adv. Mater..

[35] Wu T., Kent P.R., Gogotsi Y., Jiang D. (2022). How water attacks MXene. Chem. Mater..

[36] Chae H., Lee C., Park P., Kim I., Kim J. (2017). Synergetic effect of graphene oxide nanosheets embedded in the active and support layers on the performance of thin-film composite membranes. J. Membr. Sci..

[37] Liu S., Low Z., Hegab H.M., Xie Z., Ou R., Mohammed S., Simon G.P., Zhang X., Zhang L., Wang H. (2021). Robust hilly polyamide membrane for fast desalination. ACS Appl. Polym. Mater..

[38] Kadhom M., Albayati N., Salih S., Al-Furaiji M., Bayati M., Deng B. (2019). Role of cellulose micro and nano crystals in thin film and support layer of nanocomposite membranes for brackish water desalination. Membranes.

[39] Liu Y., Bai L., Zhu X., Xu D., Li G., Liang H., Wiesner M.R. (2020). The role of carboxylated cellulose nanocrystals placement in the performance of thin-film composite (TFC) membrane. J. Membr. Sci..

[40] Polisetti V., Ray P. (2020). Nanoparticles modified Polyacrylonitrile/Polyacrylonitrile—Polyvinylidenefluoride blends as substrate of high flux anti-fouling nanofiltration membranes. J. Appl. Polym. Sci..

[41] Xie Q., Zhang S., Hong Z., Ma H., Zeng B., Gong X., Shao W., Wang Q. (2019). A novel double-modified strategy to enhance the performance of thin-film nanocomposite nanofiltration membranes: Incorporating functionalized graphenes into supporting and selective layers. Chem. Eng. J..

[42] Kong F., Yang Z., Yue L., Chen J., Guo C. (2021). Nanofiltration membrane with substrate incorporated amine-functionalized graphene oxide for enhanced petrochemical wastewater and shale gas produced water desalination. Desalination.

[43] Tang R., Deng S., Zhao S., Shi Y., Ye J., Chen N.E., Xu Z. (2024). High permeability Performance TFC Nanofiltration Membrane with Two dimensional Nanochannels Support Layer Fabricated by Dissolving of Nanosheets. J. Environ. Chem. Eng..

[44] Wang X., Xiao Q., Wu C., Li P., Xia S. (2021). Fabrication of nanofiltration membrane on MoS2 modified PVDF substrate for excellent permeability, salt rejection, and structural stability. Chem. Eng. J..

[45] Kang Y., Jang J., Lee Y., Kim I.S. (2022). Dye adsorptive thin-film composite membrane with magnetite decorated sulfonated graphene oxide for efficient dye/salt mixture separation. Desalination.

